# Ovarian steroid cell tumor causing isosexual pseudoprecocious puberty in a young girl: an instructive case and literature review

**DOI:** 10.1186/s12902-022-00956-1

**Published:** 2022-02-16

**Authors:** Chun-Hao Chu, Wei-De Wang, Shuo-Yu Wang, Tai-Kuang Chao, Ruei-Yu Su, Chien-Ming Lin

**Affiliations:** 1 Department of Pediatrics, Tri-Service General Hospital, National Defense Medical Center, No. 325, Cheng-Kung Road, Section 2, Neihu 114, Taipei, Taiwan; 2 Department of Pediatrics, Zuoying Branch of Kaohsiung Armed Forces General Hospital, Kaohsiung, Taiwan; 3 Department of Pediatrics, Kaohsiung Medical University Hospital, Kaohsiung Medical University, Kaohsiung, Taiwan; 4 Department of Pathology, Tri-Service General Hospital, National Defense Medical Center, Taipei, Taiwan; 5 Division of Clinical Pathology, Department of Pathology, Tri-Service General Hospital, National Defense Medical Center, Taipei, Taiwan; 6 Department of Pathology and Laboratory Medicine, Taoyuan Armed Forces General Hospital, Taoyuan, Taiwan

**Keywords:** Children, Isosexual pseudoprecocious puberty, Ovarian tumors, Steroid cell tumors

## Abstract

**Background:**

Steroid cell tumors (SCTs) are very rare sex cord-stromal tumors and account only for less than 0.1% of ovarian neoplasms. SCTs might comprise diverse steroid-secreting cells; hence, the characteristic clinical features were affected by their propensity to secrete a variety of hormones rather than mass effect resulting in compression symptoms and signs. To date, ovarian SCTs have seldom been reported in children, particularly very young children; and pseudoprecocious puberty (PPP) as its unique principal manifestation should be reiterated.

**Case presentation:**

We reported a 1-year-8-month-old girl presenting with rapid bilateral breast and pubic hair development within a 2-month period. Undetectable levels of LH and FSH along with excessively high estradiol after stimulation with gonadotropin-releasing hormone (GnRH), as well as a heterogeneous mass inside left ovary shown in pelvic sonography indicate isosexual PPP. Her gonadal hormones returned remarkably to the prepubertal range the day after surgery, and histology of the ovary mass demonstrated SCTs containing abundant luteinized stromal cells.

**Conclusion:**

The case highlighted that SCTs causing isosexual PPP should be taken into consideration in any young children coexistent with rapidly progressive puberty given a remarkable secretion of sex hormones. This article also reviewed thoroughly relevant reported cases to enrich the clinical experience of SCTs in the pediatric group.

## Background

Precocious puberty is defined as the appearance of physical and hormonal signs of pubertal development before the age of 8 years in girls and 9 years in boys [1]. Etiologically, central precocious puberty (CPP) caused by early activation of the hypothalamic-pituitary-gonadal axis (HPG axis) is noticeably different from pseudoprecocious puberty (PPP) caused by endogenous sex-hormone producing tumors or exogenous hormone exposure [1]. Over 90% of the girls with CPP is idiopathic; while patients with PPP have a high risk of neoplasm existence which is a pivotal culprit for young children exhibiting rapidly progressive sexual precocity [1]. In view of this, diverse PPP-associated manifestations should be underscored to prevent delayed diagnosis and ensure the early management.

SCTs are rare tumors and account only for less than 0.1% ovarian neoplasms [2]. Histologically, they can be divided into several subtypes, such as stromal luteoma, Leydig cell tumor, or SCTs- not otherwise specified (NOS), according to their cell components [2]. Among them, SCTs-NOS make up approximately 56% of ovarian SCTs and most of the affected patients were adults with an average diagnostic age of 43 years [2]. SCTs-NOS can secrete a variety of steroid hormones; thus their clinical manifestations in adults are nonspecific and pleomorphic, including virilization or hirsutism, amenorrhea, hypercalcemia, erythrocytosis, ascites and Cushing’s syndrome in adults [2]. On the contrary, clinical experience in managing affected children was limited, which may result in delayed diagnosis and inappropriate treatment. To date, only a few children cases of SCTs-NOS have been reported, and isosexual PPP as the unique presentation has not been much emphasized.

We reported a very young girl presenting with bilateral breast and pubic hair development within a 2-month period. A heterogeneous hypoechoic cystic mass was found over her left ovary, which was histopathologically confirmed to be SCTs-NOS. After surgical removal, breast development remitted and her gonadal hormone also returned to the prepubertal range, revealing that SCTs-NOS could be effectively managed with surgical intervention upon prompt and precise diagnosis. Moreover, the present article also reviewed and integrated relevant cases from the literature to enrich the clinical experience of approaching SCTs, particularly in children.

## Case presentation

A 1-year-8-month-old girl was brought to the endocrinology outpatient clinic due to abrupt bilateral breast development and rapid growth velocity (2.0 cm/month) within 2 months. She had no perinatal or morbid records of relevance, and no use of medicine or products with phytoestrogens. On examination, her body length was 87.5 cm (90-97th percentile) and body weight was 11.3 kg (50-75th percentile). Bilateral breast showed Tanner stage III with nipple hyperpigmentation. Her pubic hair development was at Tanner stage III but there was no axillary hair development. In addition, there was no café-au-lait spots. Endocrine function test disclosed excessively high estradiol (E2) level with undetectable FSH and LH values (Table 1). Bone age study was read between 2 years old and 2 years and 6 months old at her chronological age of 1 year and 8 months.

**Table 1:** Laboratory data of patient

**Items**	**Value**	**Normal Range**		**SI units**
**Baseline data**				
E2	2420.9	< 18.4		pmol/L
FSH	< 0.10	< 0.1–7.1		IU/L
LH	< 0.10	< 0.5		IU/L
FT4	13.1	11.5–22.9		pmol/L
T4	77.7	69.3–159.6		nmol/L
TSH	0.87	0.25–5.00		mIU/L
IGF-1	19.6	7.2–31.0		nmol/L
Na^+^	139	136–145		mmol/L
K^+^	4.7	3.5–5.1		mmol/L
Cl^−^	110	98–107		mmol/L
**Tumor markers**				
AFP	3.02	0.0–10.0		μg/L
CEA	0.99	0.0–5.0		μg/L
CA-125	24.25	0.0–35.0		kU/L
CA19–9	6.54	0.0–37.0		kU/L
β-hCG	< 0.04	0.0–0.10		IU/L
**Gonadotropin-releasing hormone stimulation test**				
Time mins	FSH IU/L	LH IU/L	E2 pmol/L	Testosterone nmol/L
−30	< 0.1	< 0.1	1859.5	< 0.1
0	< 0.1	< 0.1		
30	< 0.1	< 0.1		
60	< 0.1	< 0.1	1796.4	< 0.1
90	< 0.1	< 0.1		
120	< 0.1	< 0.1	1724.4	< 0.1

*Abbreviation*: *AFP* Alpha-fetoprotein, *β-HCG* β-subunit human chorionic gonadotropin, *CA-125* Cancer antigen 125, *CA19–9* Carbohydrate antigen 19–9, *E2* Estradiol, *FSH* Follicle-stimulating hormone, *FT4* Free thyroxine, *IGF-1* Insulin-like growth factor 1, *LH* Luteining hormone, *Na*^+^, sodium, *K*^+^ Potassium, *Cl*^−^ Chloride, *T4* Thyroxine, *TSH* Thyroid-stimulating hormone

**Fig. 1:**
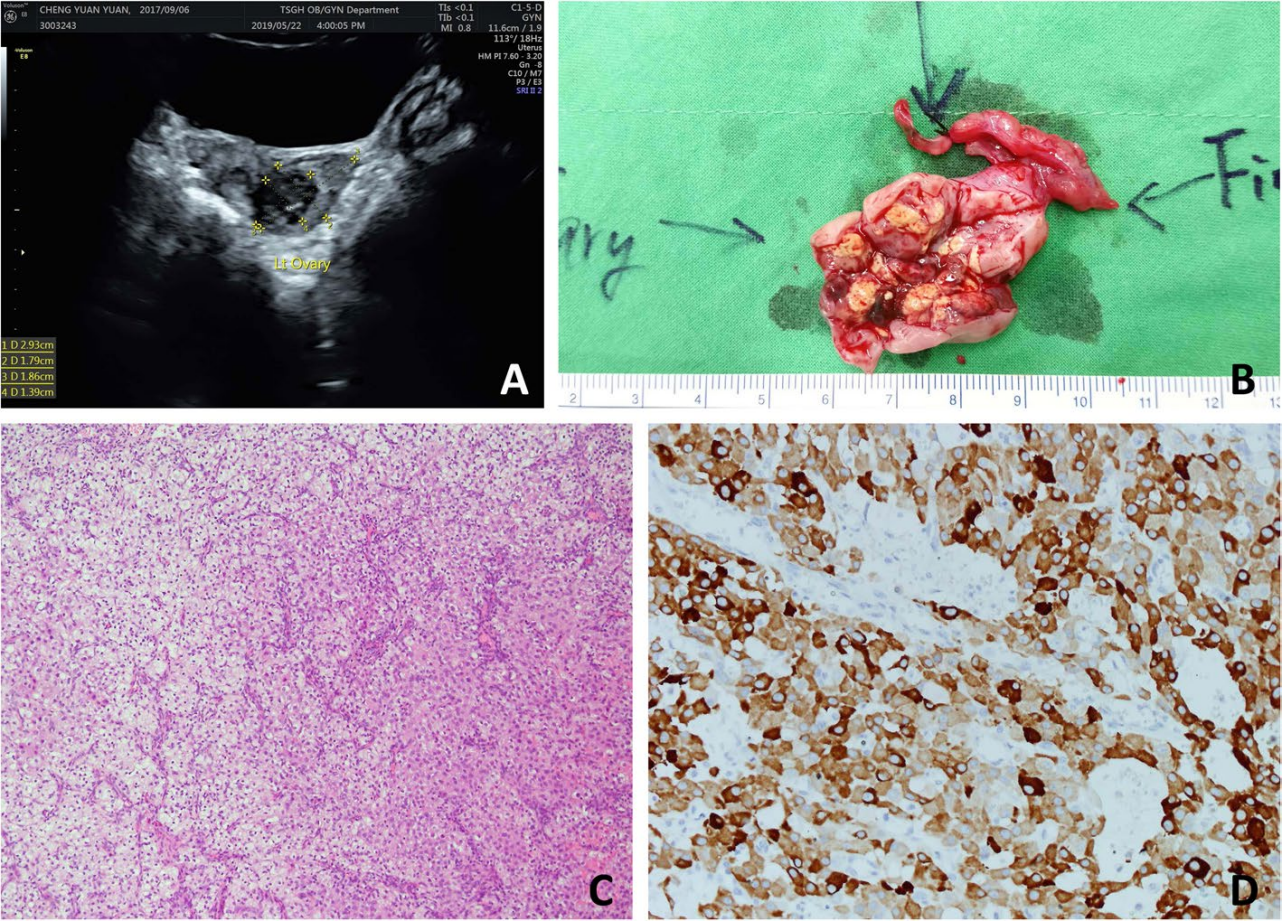
Clinical image of patient and gross and histological features of SCTs. **A**. Ultrasound showed left ovary of 2.93 × 1.79 cm in size with a heterogeneous hypoechoic cystic mass of 1.86 × 1.39 cm in size inside. **B**. Circumscribed tumor with golden-yellowish cut surface and hemorrhagic content. **C**. Tumor cells showed two cell populations of clear (left-upper) and eosinophilic cytoplasm (right-lower). **D**. Immunohistochemical features of SCTs. Positive alpha-inhibin stain, indicating a sex cord-stromal tumor. (HE, original magnification: C × 200; D × 400)

She was then admitted for further evaluation due to precocious puberty. Gonadotropin-releasing hormone (GnRH) stimulation test revealed complete suppression of baseline and peak values of LH and FSH (all < 0.1 IU/L), while baseline and peak values of E2 were 1859.5 pmol/L and 1796.4 pmol/L, respectively (Table 1), implying estrogenic development without activation of the HPG axis. Furthermore, serum tumor markers all disclosed normal. Pelvic ultrasound showed uterus size of 3.57 × 1.47 × 1.96 cm (estimated volume 5.38 cm^3^; normal: 1.05 cm^3^ on average). Right ovary was 0.96 × 0.50 cm in size with few small follicles, and left ovary was 2.93 × 1.79 cm in size with a heterogeneous hypoechoic cystic mass of 1.86 × 1.39 cm in size inside (Fig. 1A). Therefore, she was diagnosed as isosexual PPP on the basis of suppressed gonadotropin response to GnRH stimulation test and left ovarian mass lesion. Laparoscopic-assisted left salpingo-oophorectomy was performed. Grossly, the tumor was circumscribed and the cut surface reveals nodularity (Fig. 1B). Its color was golden-yellowish with hemorrhagic content. Microscopically, the tumor cells were polygonal with abundant cytoplasm ranging from vacuolated (lipid-rich) to eosinophilic (lipid-poor) (Fig. 1C). The nuclei were typical round with a prominent central nucleolus. The stroma ranged from scant to prominent with fibrous bands and conspicuous vasculature. Immunohistochemically, the tumor cells showed positive staining for alpha-inhibin (Fig. 1D) and adipophilin (not shown), confirming its nature of a sex cord-stromal tumor with steroid secreting.

One day after operation, baseline gonadal function still showed suppressed levels of gonadotropin (both FSH and LH < 0.10 IU/L), but E2 value was undetectable dramatically (< 18.4 pmol/L). Five months later, physical examination revealed bilateral breast development at Tanner stage II and disappearance of pubic hair. Follow-up pelvic ultrasound revealed a shrunk uterus of size 2.41 × 1.15 × 1.49 cm (estimated volume 2.16 cm^3^), no specific findings in left ovary, and right ovary 1.25 × 1.01 cm with few antral follicles, indicating salient improvement of pubertal progression after resection of the ovarian tumor.

## Discussion and conclusions

Ovarian SCTs are uncommon tumors first brought up by Heyes et al. in 1987, which could occur at any age even mostly found in adults [2]. To date, children cases of ovarian SCTs were scanty and their associated presentations had not been stressed, which might lead to delayed diagnosis in such young patients. Given that SCTs comprise diverse cells secreting steroid hormones, their clinical features are usually in line with secretory hormones rather than tumor mass effect. In view of this, high levels of 17-hydroxyprogesterone (17-OHP), androstenedione, and testosterone could be detected in patients with virilization and hirsutism, while increased values of E2 and cortisol were associated with isosexual PPP and Cushing’s syndrome, respectively [2]. Although adult women accounted for most reported SCTs and virilization was thought to be the most common symptom, the lack of rich clinical experience and the low awareness of isosexual PPP in children might contribute to unnecessary examination and parental anxiety. Herein, we present the youngest girl in current literature exhibiting early breast development before 2 years old, and finally diagnosed with SCTs causing isosexual PPP based on suppressed gonadotropin levels on GnRH test and typical histological findings. This case highlighted the discrepancy of clinical manifestations between adults (mainly virilization) and very young children (early feminization). However, further studies on more cases of different ages are warranted to confirm our aforementioned findings.

Among SCTs adults, more than half of them presented with hyperandrogenic signs such as hirsutism, acne, deepened voice, clitoromegaly, amenorrhea, and infertility [3]. Only a few remaining cases exhibited hyperestrogenism in terms of menorrhagia, postmenopausal bleeding, sometimes even endometrial cancer [3]. Nevertheless, reported cases in children were so scarce that their typical features remained elusive. For better understanding, previously reported children cases of SCTs (*n* = 15) were thoroughly reviewed (Table 2) [2, 4–15]. After excluding the three cases without documented clinical manifestation and one case presenting only with mass effect, a total of 12 patients including the present case were analyzed (Table 3) [4–8, 10–15]. Most of them showed heterosexual precocity (66%) with symptoms of virilization (50%), hirsutism (25%), amenorrhea (17%), hypertrichosis (17%), facial acnes (17%) and temporal balding (8%). On the contrary, isosexual precocity accounted for only 33% of all cases and the predominant symptoms were early breast development (33%), followed by vaginal bleeding (25%) and nipple pigmentation (8%). Other non-specific symptoms irrelevant to sex hormone included Cushing’s syndrome (33%) and hypertension (17%)(Table 3) [4–8, 10–15]. Herein, improved understanding of aforementioned features will add a new dimension to the precise management of ovarian SCTs.

**Table 2:** Comparison of the clinical features between reported SCTs patients and current case

No.	Study	Age (yr)	IP/HP	Clinical presentation	Gonadal function	Uterine size (cm)	Tumor size (cm)	Pathology	Tx	RT^**c**^
**Isosexual precocity**										
1	Present case	1	IP	Breast development Nipples hyperpigmentation Pubic hair growth	FSH < 0.1 IU/L^a^ LH < 0.1 IU/L^a^ E2 2420.9 pmol/L^a^	3.57 × 1.47 × 1.96	4.2 × 2.5 × 1.4	Polygonal tumor cells with abundant cytoplasm ranging from vacuolated (lipid-rich) to eosinophilic (lipid-poor)	SO	5 months
2	Haroon et al., 2015 [4]	3	IP	Breast development Vaginal bleeding	NA	NA	7.0	NA	SO	NA
3	Lin et al., 2000 [5]	3	IP	Breast development Pubic hair growth Vaginal bleeding	FSH < 1.0 IU/L^a^ LH < 0.6 IU/L^a^ E2 348.8 pmol/L^a^ Testosterone 1.2 nmol/L^a^ 17-OHP 3.9 nmol/L DHEA-S 3.1 μmol/L	6.2 × 3.2 × 2.2	4.4 × 4.1 × 3.2	NA	Resection of ovary	6 months
4	Lee et al., 2011 [6]	8	IP	Breast development Pubic hair growth Vaginal spotting Hypertension	FSH < 0.1 IU/L^a^ LH 0.29 IU/L^a^ E2 1066.1 pmol/L^a^ Aldosterone 2.3 nmol/L Angiotensin 124.0 pmol/L	NA	5.1 × 4.0	The ovarian tumor had sheets of clear or eosinophilic cells surrounded with a delicate fibrous stroma.	SO	16 months
**Heterosexual precocity**										
5	Hellyanti et al., 2021 [7]	2	HP	Cushing’s syndrome Virilization Hypertension	Cortisol 733.89 nmol/L Normal lutropin, gonadotropin and AFP	NA	5.0 × 4.2 × 3.5	The tumor comprised polygonal cells with distinct cellular borders and abundant, clear-to-granular eosinophilic cytoplasm.	SO	NA
6	Yoshimatsu et al., 2020 [8]	4	HP	Cushing’s syndrome Virilization	Testosterone 8.4 nmol/L^b^ ACTH 0.3 pmol/L Cortisol 593.1 nmol/L LDH 6.07 ukat/L NSE 36.6 μg/L	NA	8.0 × 5.0 × 5.0	The tumor was composed of both eosinophilic and vacuolated cytoplasm.	SO and C/T	4 months
7	Qian et al., 2016 [9]	5	NA	Abdominal pain Vomiting	AFP 1.15 μg/L β-HCG < 0.10 IU/L CA-125 37.71 kU/L CA19–9 30.25 kU/L	NA	8.0 × 4.0 × 7.0	The tumor was composed of cells with abundant eosinophilic to clear cytoplasm and round nuclei with prominent nucleoli	SO and C/T	No recurrence within 5 years
8	Gupta et al., 2008 [10]	5	HP	Cushing’s syndrome Facial acnes Hirsutism Hypertrichosis	E2 275.4 pmol/L^b^ Testosterone 9.2 nmol/L^b^ Progesterone 46.1 nmol/L Cortisol 913.1 nmol/L	NA	NA	The cells had abundant eosinophilic cytoplasm and were strongly positive for inhibin immunostain.	Tumor excision	4 months
9	Sawathiparnich et al., 2009 [11]	6	HP	Cushing’s syndrome	Testosterone 1.9 nmol/L^b^ ACTH 88.6 pmol/L Cortisol 1073.9 nmol/L DHEA-S 4.2 μmol/L CA-125 95.68 kU/L NSE 404.8 μg/L	NA	7 × 6 × 5	The ovarian tissue was replaced by nests or cords of round to polygonal cells with eosinophilic cytoplasm, mild nuclear atypia, and frequent intranuclear inclusions.	SO	8 months
10	Harris et al., 1991 [12]	8	HP	Virilization	NA	NA	NA	NA	Tumor excision	NA
11	Yılmaz-Ağladıoğlu et al., 2013 [13]	13	HP	Virilization	FSH 4.7 IU/L^a^ LH 1.7 IU/L^a^ E2 154.2 pmol/L^a^ Testosterone 5.1 nmol/L^a^ 17-OHP 58.4 nmol/L ACTH 1.6 pmol/L DHEA-S 3.0 μmol/L	6.9 × 2.4 × 0.9	2.3 × 2.2	Moderately pleomorphic neoplasm without mitoses or necrosis, which was surrounded by a fibrous capsule with the intact surrounding ovarian tissue	SO	6 months
12	Boyraz et al., 2013 [14]	16	HP	Amenorrhea Virilization	Testosterone 3.3 nmol/L^b^ LDH 5.2 ukat/L	NA	6 × 4 × 3.3	Minor fibromatous component and areas of hyalinization were also present.	Ovarian cystectomy	4 months
13	Ding and Hsu., 2007 [15]	16	HP	Amenorrhea Facial hirsutism Temporal balding Virilization	NA	NA	5.7 × 6.3 × 5.5	NA	Ovarian cystectomy	NA
14–16	Hayes and Scully, 1987 [2]	2–15 (3 case)	IP/HP	NA	NA	NA	NA	NA	NA	NA

*Abbreviation*: *17-OHP* 17α-OH Progesterone, *ACTH* Adrenocorticotropic hormone, *AFP* Alpha-fetoprotein, *β-HCG* β-subunit Human chorionic gonadostropin, *C/T* Chemotherapy, *CA-125* Cancer antigen 125, *CA19–9* Carbonhydrate antigen 19–9, *DHEA-S* Dehydroepiandrosterone sulfate, *E2* Estradiol, *FSH* Follicle-stimulating hormone, *HP* Heterosexual precocity, *IP* Isosexual precocity, *LDH* Lactate dehydrogenase, *LH* Luteining hormone, *NA* Non-available, *NSE* Neuron Specific Enolase, *SO* Salpingo-oophorectomy, *Tx* Treatment

^a^Data via GnRH stimulation test

^b^Unstimulated data

^c^*RT* Remission time: define as the time from initial clinical symptoms to completely remission after operation

**Table 3:** Clinical presentation among SCTs cases

Symptoms	Present case	Hellyanti et al., 2021 [7]	Yoshimatsu et al., 2020 [8]	Haroon et al., 2015 [4]	Yılmaz-Ağladıoğlu et al., 2013 [13]	Boyraz et al., 2013 [14]	Lee et al., 2011 [6]	Sawathiparnich et al., 2009 [11]	Gupta et al., 2008 [10]	Ding and Hsu., 2007 [15]	Lin et al., 2000 [5]	Harris et al., 1991 [12]	Total case (***N*** = 12)
Isosexual precocity	**+**			**+**			**+**				**+**		4/12 **(33%)**
Heterosexual precocity		**+**	**+**		**+**	**+**		**+**	**+**	**+**		**+**	8/12 **(66%)**
Breast Development	**+**			**+**			**+**				**+**		4/12 **(33%)**
Pubic hair growth	**+**	**+**					**+**				**+**		4/12 **(33%)**
Vaginal bleeding				**+**			**+**				**+**		3/12 **(25%)**
Nipples pigmentation	**+**												1/12 **(8%)**
Virilization		**+**	**+**		**+**	**+**				**+**		**+**	6/12 **(50%)**
Amenorrhea						**+**				**+**			2/12 **(17%)**
Hirsutism		**+**							**+**	**+**			3/12 **(25%)**
Facial acnes		**+**							**+**				2/12 **(17%)**
Hypertrichosis		**+**							**+**				2/12 **(17%)**
Temporal balding										**+**			1/12 **(8%)**
Cushing’s syndrome		**+**	**+**					**+**	**+**				4/12 **(33%)**
Hypertension		**+**					**+**						2/12 **(17%)**

Of note is that all reviewed cases aged less than 3 years presented with isosexual precocity except case No. 5, implying the younger the patient, the higher the possibility isosexual PPP occurrence [4–7]. Even though functioning follicular cysts are the most common cause of PPP in girls [16], this cause was excluded in our case in view of a heterogeneous hypoechoic cystic mass of ovary. Furthermore, no café-au-lait skin spots also did not support the diagnosis of McCune-Albright syndrome [17]. Therefore, a comprehensive differential diagnosis is still crucial to rule out the rare etiology such as SCTs in extremely young children. On the other hand, the patients with SCTs could present with androgenization or estrogenization, but masculinization is still the predominant symptom, especially in adults. These findings can be accounted for by the higher testosterone than E2 levels observed in a large proportion of our reviewed cases (Table 2) [2, 4–15]. In contrast, the present young girl had suppressible serum LH and FSH levels along with extremely high E2, indicating inactivation of the HPG axis and also echoing hypersecretion of estrogen caused by SCTs.

In addition to clinical manifestations and endocrine data, image studies such as sonography, computed tomography (CT), and magnetic resonance imaging (MRI) also played a pivotal role in the diagnosis of SCTs [18]. Although typical characteristics of CT and MRI are divergent depending on lipid components and fibrous stroma, SCTs usually revealed intense enhancement reflecting hypervascularity and hypointense nodular wall attributed to lipid contents [18]. Pelvic ultrasound could disclose a well-defined echogenic mass over ovaries [19]. As for immunopathology, SCTs revealed tumor cells with both eosinophilic and vacuolated cytoplasm, surrounded with fibrous stroma, as well as positive staining for alpha-inhibin and adipophilin [2]. In the present case, a heterogeneous hypoechoic cystic mass over left ovary was detected via pelvic sonography, which microscopically revealed multiple composition of lipid, fibrous tissue and vessels, as well as positive staining for alpha-inhibin and adipophilin along with clear and eosinophilic cytoplasm in the tumor specimen. SCTs could become malignant and subsequent adjuvant chemotherapy might be necessary after surgery [8, 20]; nonetheless, most of the cases had significantly good outcomes with marked decrease in sex hormones the first day after surgical resection as in our case. As expected, clinical symptoms of SCTs could remit a few months later. In summary, SCTs are rare tumors but usually benign [3, 8], and could be effectively managed with surgical intervention when prompt and precise diagnosis was made.

The present case highlighted the distinctive feature of isosexual PPP caused by SCTs in children especially those younger than 3 years of age, which was notably different from adults mainly presenting with virilization. Although the most common etiology of isosexual precocity is CPP, a high index of suspicion of peripheral lesions and detailed endocrine function tests are important for early diagnosis and treatment of rare SCTs.

## Data Availability

All the data generated and/or analyzed during this study are included in this published article.

## Abbreviations

17-OHP: 17-hydroxyprogesterone
ACTH: Adrenocorticotropic hormone
AFP: Alpha-fetoprotein
β-HCG: β-subunit human chorionic gonadotropin
CA-125: Cancer antigen 125
CA19–9: Carbohydrate antigen 19–9
CPP: Central precocious puberty
C/T: Chemotherapy
Cl-: Chloride
CT: Computed tomography
DHEA-S: Dehydroepiandrosterone sulfate
E2: Estradiol
FSH: Follicle-stimulating hormone
GnRH: Gonadotropin-releasing hormone
HP: Heterosexual precocity
HPG axis: Hypothalamic-pituitary-gonadal axis
IFG-1: Insulin-like growth factor 1
IP: Isosexual precocity
LDH: Lactate dehydrogenase
LH: Luteining hormone
MRI: Magnetic resonance imaging
NSE: Neuron specific enolase
NOS: Not otherwise specified
K +: Potassium
PPP: Pseudoprecocious puberty
SO: Salpingo-oophorectomy
Na +: Sodium
SCT: Steroid cell tumor
FT4: Free thyroxine
T4: Thyroxine
TSH: Thyroid-stimulating hormone
